# Reduction of Long-Term Disability in Inflammatory Polyarthritis by Early and Persistent Suppression of Joint Inflammation: Results From the Norfolk Arthritis Register

**DOI:** 10.1002/acr.20453

**Published:** 2011-07

**Authors:** Carlo A Scirè, Suzanne M M Verstappen, Hoda Mirjafari, Diane K Bunn, Mark Lunt, Carlomaurizio Montecucco, Ian N Bruce, Deborah P M Symmons

**Affiliations:** 1 University of ManchesterManchester, UK; 2 University of ManchesterManchester, UK; 3 Norfolk Arthritis Register, Norfolk and Norwich University HospitalNorwich, UK; 4 IRCCS Policlinico San Matteo Foundation, University of PaviaPavia, Italy

## Abstract

**Objective:**

To test the predictive ability of remission in terms of long-term disability in patients with recent-onset inflammatory polyarthritis (IP).

**Methods:**

Consecutive patients with early IP, recruited between 1990 and 1994 (first cohort) and 2000 and 2004 (second cohort), were included in this study. Remission was defined as the absence of clinically detectable joint inflammation on a 51–joint count. In additional analyses, less stringent definitions of remission were used based on the 40– and 28–joint counts. Remission was assessed at 1, 2, and 3 years after inclusion. A 5-year Health Assessment Questionnaire score ≥1 (moderate disability) was chosen as the primary outcome measure.

**Results:**

A total of 841 and 498 patients from the first and second cohorts, respectively, completed 5 years of followup. In the first cohort, patients with at least 1 episode of remission had lower odds of 5-year disability (odds ratio [OR] 0.26, 95% confidence interval [95% CI] 0.17–0.41). The number of times in remission correlated with the odds of disability, with a mean decrease in the probability of disability of ∼64% for each additional time point in remission (OR 0.38, 95% CI 0.28–0.52). The time until first remission was not associated with functional disability. Remission according to less stringent criteria showed a weaker protection against future disability. Similar results were found in the second cohort.

**Conclusion:**

Patients with IP achieving a state of sustained remission early are less likely to show long-term deterioration of function compared with patients who do not achieve remission. The most persistent remission under the most stringent definition of remission has the lowest probability of long-term disability.

## INTRODUCTION

The aim of current treatment of inflammatory polyarthritis (IP) is to achieve early and sustained control of joint inflammation in order to provide relief of symptoms and to prevent long-term detrimental outcomes such as joint damage, functional disability, and increased mortality (1).

The relevance of aiming to suppress joint inflammation primarily arises from pathophysiologic studies in patients with chronic arthritis that clearly indicate the central role of chronic inflammation in producing factors such as proinflammatory cytokines that lead to structural deterioration of joints (2). Also, imaging studies in rheumatoid arthritis (RA) suggest that the progression of joint damage is related to the amount of synovial inflammation, even in subclinical disease (3,4). Several clinical studies have shown that early and/or aggressive therapeutic interventions lead to complete control of disease activity, i.e., clinical remission, in an increasing proportion of patients with early RA (5–10). Furthermore, these new treatment strategies are able to modify the clinical course of the disease in terms of clinical outcome, joint damage, and functional disability (11–13).

Combining biologic and clinical data, aiming for remission seems to be the most effective strategy to prevent long-term detrimental outcomes. Unfortunately, evidence to support this simple statement from routine clinical practice is lacking for some practical and conceptual reasons. First, as different measures are in use for assessing disease activity in RA, a consensus definition of clinical remission in RA is currently unavailable (14). Second, it is still unclear whether remission status might truly predict relevant long-term outcomes such as future disease activity, structural damage, functional disability, and mortality.

These issues have been clearly outlined in a recent systematic review (15). Consistent data support that remission, regardless of the applied definition, is longitudinally related to radiologic progression. Interestingly, there is only limited evidence that remission might be longitudinally associated with a better functional outcome. More recently, Tanaka et al (16), studying a large cohort of patients with established RA, identified an inverse correlation between an index of cumulative remission over time and the development of functional disability by the last observation.

However, it is still unclear what the actual association is between remission and long-term disability, what the effect is of a persistent status of remission on long-term disability, and what the influence is of using different definitions of remission on long-term disability.

The Norfolk Arthritis Register (NOAR) is a unique inception cohort of patients with early IP who are followed longitudinally using standardized protocols (17). Using patients recruited by the NOAR, we therefore sought to investigate the association between the time from symptom onset until the first remission, the status of sustained remission, and the use of different definitions of remission during the first 3 years with functional disability at 5 years after inclusion in the register.

## SUBJECTS AND METHODS

### Study design, setting, and participants

Since 1990, patients with early IP have been recruited to the NOAR, a large primary care–based inception cohort in the east of the UK. A detailed description of this register has been reported elsewhere (17). Briefly, consecutive cases of IP are notified through general practitioners or attendance at hospital rheumatology clinics within this catchment area. The notification criteria are adults ages ≥16 years at symptom onset and swelling of at least 2 joints that has persisted for at least 4 weeks. Individuals referred to the register were subsequently assessed by a metrologist within the next couple of weeks, in which steroid injections could have been given or disease-modifying antirheumatic drugs (DMARDs) started. Those who were diagnosed by a hospital consultant with a condition other than RA, IP, psoriatic arthritis, or viral arthritis, which accounted for their joint symptoms, were excluded. For the present study, we included only patients recruited between 1990 and 1994 (cohort 1) and between 2000 and 2004 (cohort 2) because in these two cohorts, patients were assessed at baseline and at 1, 2, 3, and 5 years after inclusion in the register. This study was conducted with the approval of the Norfolk and Norwich University Hospital Local Research Ethics Committee. All subjects gave written consent.

### Demographic and clinical assessments

At baseline, patients were assessed by a research nurse using a structured interview and clinical examination. The baseline data included demographics (age at onset of symptoms, sex, and time from symptom onset to notification to the NOAR), comorbidities, and previous/current use of DMARDs. Start and stop dates of DMARDs, including oral corticosteroids, were also collected at each followup visit. At baseline and followup, clinical assessments included the number of swollen and tender joints (based on 51– and 28–joint counts). Blood samples were taken to determine rheumatoid factor (RF) and C-reactive protein (CRP) levels (18,19). The 28-joint Disease Activity Score (DAS28) using the CRP level (20) was then calculated. The American College of Rheumatology (ACR) 1987 criteria for RA (21) were applied cross-sectionally at baseline.

### Remission

Remission was primarily defined as no swollen and no tender joints on examination of 51 joints (definition 1). To make results more comparable with other studies, remission was also defined as no swollen and no tender joints on examination of 40 of 51 assessed joints (excluding neck, hips, and proximal interphalangeal joints) (definition 2). In addition, less stringent definitions of remission were applied: no swollen and no tender joints based on the 28–joint count (definition 3), and ≤1 swollen joint and ≤1 tender joint based on the 28–joint count (definition 4). For each of these definitions of remission, different remission states were defined: 1) “remission ever”: at least one assessment in remission within the first 3 years, 2) “time to remission”: year of the first assessment in remission within the first 3 years, 3) “remission score”: times in remission within the first 3 years, and 4) “rewarded score”: remission score plus an extra point for subsequent remissions and for remission at the last observation (third-year assessment) (22,23).

Patients showing a self-limiting IP, defined as a persistent absence of joint swelling and tenderness lasting from the first to the third year in the absence of DMARD treatment, were classified as being in “natural remission” and were not included in the analysis.

### Functional disability

We included all patients who completed the British version of the Health Assessment Questionnaire (HAQ) at baseline and annually thereafter until 5 years of followup (24). The main outcome of this study was the 5-year HAQ score, dichotomized into absence versus presence of functional disability (HAQ score ≥1) (25–29).

### Statistical analysis

Baseline differences between the two cohorts were tested using Wilcoxon's rank sum test for continuous variables and the chi-square test for categorical variables. The effect of remission on the development of long-term disability was analyzed using logistic regression models for the first and second cohort separately, and odds ratios (ORs) with 95% confidence intervals (95% CIs) are shown accordingly. Primary analysis investigated the association between remission 1 and disability at 5 years, including each of the following individual remission states as independent variables in separate models: 1) “remission ever,” 2) “time to remission,” 3) “remission score,” and 4) “rewarded score.” The same analyses were repeated adjusting for potential baseline confounders, coded as follows: sex, RF (≥1:40), ACR criteria for RA, and previous and/or concurrent treatment with DMARDs and/or glucocorticoids as categorical variables, and symptom duration, age in decades, and DAS28 and HAQ scores as continuous variables. All confounders were entered and retained in the model regardless of their statistical significance. Effect modification was explored by fitting interactions in logistic models. The accuracy of the model was analyzed using the area under the receiving operating characteristic curve (AUC).

In secondary analysis, a second, third, and fourth set of logistic regression models similar to the first one were generated, including remission variables (ever in remission, time to remission, remission score, and rewarded score), according to definitions 2, 3, and 4, respectively.

A total of 1,098 patients from the first cohort and 696 from the second cohort were eligible for the study. Of these patients, 90 (8.1%) from the first cohort and 57 (8.1%) from the second cohort died during the followup period under study; 161 (14.6%) and 121 (17.3%) subjects, respectively, were lost to followup; and 3 (0.3%) and 15 (2.2%) patients, respectively, who fulfilled the criteria for “natural remission” during the followup were excluded. The study sample finally comprised 844 and 503 subjects from the first and second cohorts, respectively. HAQ score was available for 841 and 498 subjects, respectively. Of the 841 and 498 evaluable subjects from the first and second cohorts with HAQ scores, respectively, 729 (86.7%) and 452 (90.8%), respectively, had complete data on joint counts at every time point (1.3% missing at 1 year, 6.5% missing at 2 years, and 8% missing at 3 years). Due to the inclusion of several confounders, the number of subjects with complete data available for the analyses fell to 583 (69.0%) and 326 (64.6%), respectively. To increase the precision of our analyses, missing data on confounders and joint counts were therefore imputed using switching regression, an iterative multivariable regression technique that retains an element of random variation in the estimates (30). Using multiple imputation of remission variables and confounders, 841 and 498 subjects, respectively, were finally available for all of the adjusted analyses. All analyses were conducted using Stata, version 10 (StataCorp).

## RESULTS

Baseline characteristics of the study population are summarized in Table 1. In general, the first cohort comprised patients with a shorter disease duration, higher disease activity and disability, and lower prevalence of treatment at the time of inclusion in the NOAR. There were both quantitative and qualitative differences in the therapeutic management of the two cohorts. A total of 44.6% of patients in the first cohort and 71.9% in the second cohort were ever treated with DMARDs during followup. In the first cohort, the use of DMARDs was less persistent (median months receiving DMARDs 0 [interquartile range (IQR) 0–47 months] versus 51 [IQR 0–60 months]). Furthermore, DMARD treatment differed qualitatively between the two cohorts, with a lower use of methotrexate ever in the first cohort (20.3% versus 55.2%). These results reflect the changing management of RA and IP during the last two decades.

**Table 1:** Baseline characteristics of cohort 1 (1990–1994) and cohort 2 (2000–2004)*

	First cohort (1990–1994)	Second cohort (2000–2004)
N	841	498
Age at symptom onset, years	54.1 (42.7–65.1)	57.3 (47.8–68.3)†
Women, no. (%)	569 (67.7)	345 (69.3)
Symptom duration, months	5.52 (2.8–10.6)	8.15 (4.50–16.3)†
Current smoker, no. (%)	213 (25.3)	102 (20.5)
Satisfied 1987 ACR criteria for RA, no. (%)	398 (47.3)	236 (47.4)‡
Rheumatoid factor positive, no. (%)§	208 (27.8)	153 (33.9)
Presence of nodules, no. (%)	56 (6.6)	34 (6.8)
Morning stiffness	30 (0–90)	30 (2–60)
Swollen joint count of 51	7 (2–13)	3 (1–7)†
Tender joint count of 51	8 (3–17)	4 (1–11)†
Swollen joint count of 28	5 (2–11)	2 (0–6)†
Tender joint count of 28	5 (2–11)	2 (0–7)†
CRP level, mg/liter¶	5 (0–14.0)	8.6 (2.7–20.0)†
DAS28-CRP (3)¶	3.94 (2.9–5.0)	3.53 (2.5–4.4)†
HAQ score	0.750 (0.2–1.4)	0.875 (0.4–1.6)†
Receiving DMARDs, no. (%)	146 (17.3)	229 (45.9)‡
Receiving steroids, no. (%)	52 (6.2)	137 (27.5)‡

* Values are the median (interquartile range) unless otherwise indicated. ACR = American College of Rheumatology; RA = rheumatoid arthritis; CRP = C-reactive protein; DAS28-CRP = 28-joint Disease Activity Score using the CRP level; HAQ = Health Assessment Questionnaire; DMARDs = disease-modifying antirheumatic drugs.

† *P* < 0.05 by Mann-Whitney U test.

‡ *P* < 0.05 by chi-square test.

§ Rheumatoid factor measured on 748 and 457 subjects, respectively.

¶ CRP level measured on 688 and 420 subjects, respectively.

The prevalence of remission at each anniversary during the first 3 years of followup is shown in Table 2. There was an increased prevalence of remission in the second cohort compared to the first cohort, regardless of the applied definition of remission. The same trend was observed for the cumulative prevalence of remission (remission ever) and remission or rewarded scores. As expected, less stringent definitions led to an increasing occurrence of remission in both cohorts. As a result of this, the occurrence of remission ever ranged from 22.4% to 33.9% for the most stringent criterion (definition 1) to 46.5% to 57.7% for the most permissive one (definition 4) in the first and second cohorts, respectively. After 5 years of followup, 376 (44.7%) and 263 (52.8%) of subjects from the first and second cohorts, respectively, experienced at least moderate disability (HAQ score ≥1).

**Table 2:** Occurrence of clinical remission within the first 3 years of followup*

	Remission 1†		Remission 2‡		Remission 3§		Remission 4¶	
				
	First cohort (1990–1994)	Second cohort (2000–2004)	First cohort (1990–1994)	Second cohort (2000–2004)	First cohort (1990–1994)	Second cohort (2000–2004)	First cohort (1990–1994)	Second cohort (2000–2004)
Cross-sectional								
First year	49 (6.6)	72 (15.3)	57 (7.7)	77 (16.6)	79 (10.6)	91 (19.4)	153 (20.7)	162 (34.5)
Second year	80 (10.8)	91 (19.4)	88 (11.9)	97 (20.9)	114 (15.4)	119 (25.3)	203 (27.4)	176 (37.5)
Third year	98 (13.2)	96 (20.4)	107 (14.5)	103 (22.2)	133 (18.0)	125 (26.6)	216 (29.2)	167 (35.6)
Remission ever	166 (22.4)	159 (33.9)	182 (24.6)	167 (36.1)	221 (29.9)	202 (43.0)	344 (46.5)	271 (57.7)
Time to first remission								
First year	49 (6.6)	72 (15.3)	57 (7.7)	77 (16.6)	79 (10.6)	91 (19.4)	153 (20.7)	162 (34.5)
Second year	57 (7.7)	55 (11.7)	60 (8.1)	58 (12.5)	77 (10.4)	68 (14.5)	116 (15.7)	76 (16.2)
Third year	60 (8.1)	32 (6.8)	65 (8.8)	32 (6.9)	65 (8.8)	43 (9.1)	75 (10.1)	33 (7.0)
Remission score, median (IQR)	0 (0–0)	0 (0–1)	0 (0–1)	0 (0–1)	0 (0–1)	0 (0–1)	0 (0–1)	1 (0–2)
Rewarded score, median (IQR)	0 (0–0)	0 (0–1)	0 (0–1)	0 (0–2)	0 (0–1)	0 (0–2)	0 (0–2)	1 (0–3)

* Values are the number (percentage) unless otherwise indicated. Evaluated on complete data on remission of 738 subjects from the first cohort and 462 from the second cohort. IQR = interquartile range.

† Remission 1: 51–swollen joint count + 51–tender joint count = 0.

‡ Remission 2: 40–swollen joint count + 40–tender joint count = 0.

§ Remission 3: 28–swollen joint count + 28–tender joint count = 0.

¶ Remission 4: 28–swollen joint count ≤1 and 28–tender joint count ≤1.

### Primary analysis in the first cohort

In the first cohort, subjects who had experienced at least one period of remission according to definition 1 within the first 3 years of followup showed a 75% relative reduction in the odds of being moderately disabled after 5 years (OR 0.25, 95% CI 0.17–0.37). This reduction was still ∼70% after adjusting for baseline confounders (OR 0.33, 95% CI 0.20–0.54) and optimizing information using multiple imputations of missing data (adjusted OR 0.26, 95% CI 0.17–0.41) (Table 3). The same trend was seen for the imputed/adjusted data and therefore, these ORs are shown for all analyses. No significant interactions were found between remission variables and possible baseline confounders.

**Table 3:** Effect of remission according to different definitions of remission on 5-year moderate disability (HAQ score ≥1)*

Cohort	Remission 1, adjusted OR (95% CI)†	Remission 2, adjusted OR (95% CI)‡	Remission 3, adjusted OR (95% CI)§	Remission 4, adjusted OR (95% CI)¶
Ever in remission				
1990–1994	0.26 (0.17–0.41)	0.31 (0.20–0.46)	0.23 (0.15–0.34)	0.31 (0.21–0.44)
2000–2004	0.27 (0.16–0.44)	0.31 (0.19–0.51)	0.39 (0.24–0.63)	0.51 (0.32–0.82)
Remission score				
1990–1994	0.38 (0.28–0.52)	0.44 (0.33–0.59)	0.40 (0.30–0.52)	0.47 (0.37–0.59)
2000–2004	0.43 (0.31–0.58)	0.45 (0.34–0.61)	0.51 (0.39–0.66)	0.55 (0.44–0.70)
Time to first remission				
1 year				
1990–1994	0.28 (0.15–0.53)	0.39 (0.22–0.69)	0.27 (0.16–0.47)	0.37 (0.24–0.57)
2000–2004	0.29 (0.15–0.58)	0.31 (0.16–0.60)	0.35 (0.19–0.65)	0.45 (0.27–0.77)
2 years				
1990–1994	0.22 (0.11–0.44)	0.20 (0.10–0.40)	0.18 (0.09–0.34)	0.19 (0.10–0.36)
2000–2004	0.24 (0.11–0.53)	0.25 (0.12–0.53)	0.33 (0.16–0.66)	0.49 (0.23–1.02)
3 years				
1990–1994	0.30 (0.15–0.59)	0.33 (0.18–0.64)	0.24 (0.16–0.47)	0.30 (0.15–0.59)
2000–2004	0.26 (0.10–0.68)	0.45 (0.18–1.11)	0.63 (0.28–1.38)	0.85 (0.35–2.06)
Rewarded score				
1990–1994	0.54 (0.44–0.67)	0.60 (0.49–0.72)	0.56 (0.47–0.67)	0.62 (0.53–0.71)
2000–2004	0.57 (0.47–0.71)	0.60 (0.50–0.73)	0.65 (0.55–0.77)	0.69 (0.59–0.79)

* All analyses adjusted for age, sex, disease duration, rheumatoid factor, disease-modifying antirheumatic drug use at baseline, steroid use at baseline, baseline 28-joint Disease Activity Score, baseline HAQ score, and baseline comorbidities. Based on multiple imputation of missing data. First cohort: 841 subjects, second cohort: 498 subjects. HAQ = Health Assessment Questionnaire; OR = odds ratio; 95% CI = 95% confidence interval.

† Remission 1: 51–swollen joint count + 51–tender joint count = 0.

‡ Remission 2: 40–swollen joint count + 40–tender joint count = 0.

§ Remission 3: 28–swollen joint count + 28–tender joint count = 0.

¶ Remission 4: 28–swollen joint count ≤1 and 28–tender joint count ≤1.

The timing of the first achievement of remission within the first 3 years was not relevant in terms of future disability. Although the achievement of the first remission was significantly associated with a lower risk of disability at each time point within the first 3 years, no systematic decrease in OR was found across the increasing time to the first remission.

Analyzing the subgroup of patients receiving treatment with DMARDs during the followup, the achievement of remission at the first assessment was associated with the lowest OR of disability (time to first remission 1 year: adjusted OR 0.14, 95% CI 0.04–0.44; 2 years: adjusted OR 0.25, 95% CI 0.10–0.65; and 3 years: adjusted OR 0.22, 95% CI 0.09–0.55). Conversely, in patients untreated with DMARDs, the achievement of remission at the first assessment was associated with the higher OR of disability (time to first remission 1 year: adjusted OR 0.57, 95% CI 0.25–1.30; 2 years: adjusted OR 0.18, 95% CI 0.06–0.58; and 3 years: adjusted OR 0.40, 95% CI 0.13–1.13).

The number of assessments in remission (remission score) was proportional to the odds of moderate disability, with a mean decrease in the probability of moderate disability of ∼64% for each time point in remission (adjusted OR 0.38, 95% CI 0.28–0.52). The effect of remission score was linearly associated with the outcome, leading to a proportional reduction of the odds of disability for each increasing point of remission score (1-point score: adjusted OR 0.36, 95% CI 0.23–0.58; 2-point score: adjusted OR 0.14, 95% CI 0.05–0.36; and 3-point score: adjusted OR 0.07, 95% CI 0.00–0.66). Similar results were obtained for the rewarded score. Using the rewarded score instead of the simple remission score did not significantly modify the accuracy of the regression model (AUC 0.824, 95% CI 0.803–0.846 versus AUC 0.824, 95% CI 0.802–0.846).

### Primary analysis in the second cohort

The analysis of the second cohort confirmed the longitudinal effect of a remission status achieved within the first 3 years on 5-year moderate disability (Table 3). In the unadjusted analyses, the effect of remission on 5-year HAQ score was slightly higher in the second cohort compared to the first cohort (OR 0.15, 95% CI 0.10–0.24 versus OR 0.25, 95% CI 0.17–0.37), but this difference disappeared after adjusting for confounders and using multiple-imputed data sets (adjusted OR 0.26, 95% CI 0.17–0.41 versus OR 0.27, 95% CI 0.16–0.44). The accuracy of the models fitted on the second set of data was as good as that observed in the first one (AUC 0.818, 95% CI 0.795–0.840 for the adjusted model, including remission score), confirming that the observed relationship between remission and future disability is independent of the cohort and the treatment received.

### Secondary analysis

To make results more comparable with our studies, we also tested an alternative definition of remission 1 as no swollen and no tender joints on examination of 40 of 51 joints assessed. Both prevalence (agreement 98.2%) and effect measures of remission according to these two definitions were highly consistent in both cohorts (Table 3).

In order to verify the relevance of a stricter definition of remission on future disability, we tested and compared the effect of 2 other definitions that were progressively more permissive in terms of residual joint involvement (Table 3). Clinical remission, even according to the more permissive criteria, was associated with a better functional outcome. Slight differences between the two cohorts were observed. A trend to a decreasing protective effect for less stringent remission criteria was more evident in the second cohort than in the first cohort.

Since less stringent criteria also included subjects fulfilling more stringent criteria, we used contrasts to separate the single effect of each definition (Table 4). Overall, subjects in remission according to the most stringent criterion (definition 1) showed the lowest probability of 5-year disability.

**Table 4:** Specific effect of remission according to different definitions of remission on 5-year moderate disability (HAQ score ≥1)*

Ever in remission according to definition	First cohort (1990–1994), adjusted OR (95% CI)	Second cohort (2000–2004), adjusted OR (95% CI)	All subjects, adjusted OR (95% CI)
No remission definitions	1	1	1
Remission 4 definition	0.84 (0.46–1.55)	1.17 (0.55–2.49)	0.95 (0.59–1.51)
Remission 3 and 4 definitions	0.22 (0.10–0.47)	1.16 (0.52–2.56)	0.46 (0.28–0.77)
Remission 1, 3, and 4 definitions	0.23 (0.15–0.35)	0.28 (0.16–0.48)	0.24 (0.17–0.34)

* Adjusted for age, sex, disease duration, rheumatoid factor, disease-modifying antirheumatic drug use at baseline, steroid use at baseline, baseline 28-joint Disease Activity Score, baseline HAQ score, and baseline comorbidities. Based on imputed missing data. First cohort: 841 subjects, second cohort: 498 subjects. HAQ = Health Assessment Questionnaire; OR = odds ratio; 95% CI = 95% confidence interval; remission 4 = 28–swollen joint count ≤1 and 28–tender joint count ≤1; remission 3 = 28–swollen joint count + 28–tender joint count = 0; remission 1 = 51–swollen joint count + 51–tender joint count = 0.

In the first cohort, the subgroup of subjects in remission according to definition 3 but not definition 1 showed a benefit on future disability, while subjects in remission only according to definition 4 did not. In the second cohort, the specific effect of neither only in remission 3 nor only in remission 4 gave a significant effect on 5-year disability. Analyzing both cohorts together, a significant trend toward the higher protective effect for the more stringent criterion was found (score test for trend of odds *P* < 0.0001).

## DISCUSSION

This study sought to determine the predictive value of a pragmatic definition of remission on the development of moderate disability by 5 years in a primary care–based inception cohort of patients with IP. To our knowledge, this is the largest study investigating the relationship between clinical remission and long-term functional outcome in IP. The particular setting of the NOAR allowed us to measure the effect of remission taking into account several potential confounders, providing more precise and less unbiased measures of effect. Due to the sequential recruitment of patients we were also able to test and compare such measures of effect in two separate cohorts, increasing the generalizability of the results.

We found that the achievement of a status of remission within the first 3 years of followup was associated with a fall of approximately 70% in the odds of moderate disability after 5 years. This result is in keeping with previous findings that describe a significant difference of long-term disability measures comparing patients who went into remission with those who did not (31–33). In our analysis, we also adjusted for baseline confounders, including the HAQ and disease activity, which affect both the probability of entering into a remission status and of being disabled over time. Adjusted analyses better define the relationship between remission and disability, limiting their intrinsic association in patients with mild and nonprogressive disease.

Among subjects in clinical remission within the first 3 years, there were subjects who fulfilled the criteria of remission only once and subjects who were in remission on 2 or 3 annual followup visits. The number of times spent in remission showed a proportional effect on long-term disability, with a relative decrease of the odds of 60% for each assessment in remission within the first 3 years. As a result of this, the estimated risk of moderate disability for subjects in remission at 3 followup visits was more than 5 times lower than that of subjects who were in remission only once. Previous studies have already identified an association between a persistent status of remission and a better radiographic outcome in patients with RA (29,34). Our results support the clinical relevance of aiming to achieve a persistent status of clinical remission.

Using a rewarded score, which gives more weight to consecutive assessments and last assessment in remission, we tried to identify the effect of persistent remission (23). The lack of any advantage over remission score might be due to the small number and frequency of assessments included in our analysis rather than a true absence of difference.

In this study, we included two cohorts of patients recruited 10 years apart. We found a number of baseline and longitudinal differences. The second cohort comprised patients with a longer mean symptom duration, higher disease activity, higher baseline disability, and higher prevalence of patients already receiving treatment at the time of registration in the NOAR. On the other hand, due to the higher disease activity and severity at baseline, a larger proportion of patients from the second cohort developed at least moderate disability after 5 years. Probably due to a more intensive treatment strategy (more persistent and aggressive DMARD therapy), a higher proportion of patients from the second cohort achieved a status of clinical remission. Despite these differences, the effect of remission was highly consistent in both cohorts.

Interestingly, the time to first remission within the first 3 years did not affect the risk of 5-year disability. This is in contrast to other published studies that suggest that early intervention is associated with a lower risk of progression of joint damage in RA and disability, probably due to the earlier suppression of disease activity (11,33,35). One possible explanation for our results could be the width of the window of opportunity for the prevention of disability. Since a similar protective effect was evident for remission achieved at each time point between 1 and 3 years, we can speculate that the opportunity for preventing long-term disability is wider than 3 years. A second explanation could be the loss of the sustained effect on long-term HAQ score of an earlier clinical response, as previously reported (36), or the different effect on disease activity and disease severity components of the HAQ. A further explanation relies on the applied study design. In our observational setting that includes IP regardless of the fulfillment of RA classification criteria, disease activity and therapeutic decision are strictly interdependent, so that more severe disease is treated more intensively and less severe disease is likely to be untreated. Moreover, remission in treated patients is more likely to be more sustained between annual clinical assessments, while in untreated patients it is more likely to be a random fluctuation around a status of low disease activity. As a matter of fact, stratifying our analyses by DMARD treatment over the first 5 years, earlier remission showed a trend of association with better functional outcome in the subset of patients treated during the followup.

We also explored the impact of definitions of remission progressively more permissive in terms of residual joint inflammation. Regardless of the applied definition, we were able to detect a significant protective effect of remission on the risk of moderate disability. Investigating the specific effect of each definition of remission, we found that the greatest effect was due to the fulfillment of the most stringent definition. The additional protective effect of remission based on less stringent criteria was not consistent across the two cohorts or even no longer significant. This result is in keeping with the current belief that more stringent criteria for remission (e.g., Simplified Disease Activity Index, Clinical Disease Activity Index) could identify a more robust status of complete disease control compared with more permissive criteria (e.g., DAS28) (14,37). This explorative analysis aimed to identify a relationship between “stringency” and long-term relevant outcomes, rather than to provide a clinically applicable cutoff for predicting future disability.

This study has some limitations. Due to the study design, we were not able to apply any of the current definitions of remission (neither dimensional criteria based on specified cutoffs of continuous disease activity scores nor categorical criteria based on fulfillment of prespecified items) (14,37). Our definitions, only based on swollen and tender joint counts, clearly lack content validity, since they exclude acute-phase reactants and the patient's or assessor's reported measures. Nevertheless, the lack of complete content validity does not affect the predictive validity of our definitions of remission.

The number of patients lost to followup was acceptable for a longitudinal study (38). Subjects lost to followup but still alive after 5 years were significantly younger than those who were lost to followup, but showed similar baseline disease activity and functional disability. Conversely, subjects who died during the followup were older and showed higher disease activity and disability than completers. These differences introduce a selection bias that should be taken into account when interpreting our results.

In summary, this study demonstrates that achieving sustained remission early in the disease course of IP is associated with longitudinally better functional outcome. This result strengthens the use of remission as a relevant disease outcome to target in clinical practice. More ambitious targets, such as sustained remission and complete control of joint inflammation, will be an even better outcome to aim for in patients with IP in order to prevent future disability. These results, tested and confirmed in two separate sets of data, are likely to be generalizable to the entire population of IP.
